# DFT-D4 Insight into the Inclusion of Amphetamine and Methamphetamine in Cucurbit[7]uril: Energetic, Structural and Biosensing Properties

**DOI:** 10.3390/molecules26247479

**Published:** 2021-12-10

**Authors:** Abdelkarim Litim, Youghourta Belhocine, Tahar Benlecheb, Monira Galal Ghoniem, Zoubir Kabouche, Fatima Adam Mohamed Ali, Babiker Yagoub Abdulkhair, Mahamadou Seydou, Seyfeddine Rahali

**Affiliations:** 1 Laboratory of Sensors, Instrumentations and Process (LCIP), University of Abbes Laghrour, Khenchela 40000, Algeria; Litim.abdelkarim@univ-khenchela.dz (A.L.); Kabouche.zoubir@univ-khenchela.dz (Z.K.); 2 Department of Petrochemical and Process Engineering, Faculty of Technology, 20 August 1955 University of Skikda, El Hadaik Road, Skikda 21000, Algeria; y.belhocine@univ-skikda.dz; 3 Department of Chemistry, College of Science, Imam Mohammad Ibn Saud Islamic University (IMSIU), Riyadh 11432, Saudi Arabia; mgghoniem@imamu.edu.sa; 4 Chemistry Department, College of Science, Imam Mohammad Ibn Saud Islamic University (IMSIU), Riyadh 11432, Saudi Arabia; byabdulkhair@imamu.edu.sa; 5 Chemistry Department, Sudan University of Science and Technology (SUST), Khartoum 13311, Sudan; 6 Université de Paris, CNRS, ITODYS, F-75013 Paris, France; mahamadou.seydou@univ-paris-diderot.fr; 7 Department of Chemistry, College of Science and Arts, Qassim University, Ar Rass, Saudi Arabia

**Keywords:** cucurbit[7]uril, amphetamine, methamphetamine, inclusion complex, DFT-D4, drug sensing

## Abstract

The host–guest interactions of cucurbit[7]uril (CB[7]) as host and amphetamine (AMP), methamphetamine (MET) and their enantiomeric forms (S-form and R-form) as guests were computationally investigated using density functional theory calculations with the recent D4 atomic-charge dependent dispersion corrections. The analysis of energetic, structural and electronic properties with the aid of frontier molecular orbital analysis, charge decomposition analysis (CDA), extended charge decomposition analysis (ECDA) and independent gradient model (IGM) approach allowed to characterize the host–guest interactions in the studied systems. Energetic results indicate the formation of stable non-covalent complexes where R-AMP@CB[7] and S-AMP@CB[7] are more stable thermodynamically than R-MET@CB[7] and S-MET@CB[7] in gas phase while the reverse is true in water solvent. Based on structural analysis, a recognition mechanism is proposed, which suggests that the synergistic effect of van der Waals forces, ion–dipole interactions, intermolecular charge transfer interactions and intermolecular hydrogen bonding is responsible for the stabilization of the complexes. The geometries of the complexes obtained theoretically are in good agreement with the X-ray experimental structures and indicate that the phenyl ring of amphetamine and methamphetamine is deeply buried into the cavity of CB[7] through hydrophobic interactions while the ammonium group remains outside the cavity to establish hydrogen bonds with the portal oxygen atoms of CB[7].

## 1. Introduction

In 1887, the Romanian chemist, Lazăr Edeleanu discovered synthetic psychostimulants which are known as amphetamines [1,2,3]. This class of drugs is used as pharmacotherapy for obesity, narcolepsy and attention-deficit/hyperactivity disorder (ADHD) [4]. Despite their therapeutic benefits, amphetamines have been associated with several adult health issues and an increasing concern about the growing number of babies who are subjected to these drugs due to maternal use during the pregnancy period [5]. These stimulant drugs work by stimulating the central nervous system [6,7], they increase symptoms such as alertness and focus and decrease others such as tiredness and appetite [8]. 

As reported by the World Health Organization, amphetamine-type stimulants (ATS) belong to a class of drugs that include amphetamine (AMP), methamphetamine (MET) and some stimulants which are structurally related [9].

Methamphetamine is commonly referred to as “meth” or “crystal meth”. It is a very strong stimulant of the central nervous system (psychostimulant) which is derived from phenethylamine and amphetamine group of substances. Similarly to amphetamine, methamphetamine effects result in an increased level of wakefulness, attention and energy; however, taking high doses will expose users to higher levels of euphoria, sexual desire and self-esteem [10,11]. Methamphetamine is the most used illegal stimulant after Cannabis [12]. It is also the most produced among ATS around the world [13].

Immunochromatography and enzyme-linked immunosorbent assay (ELISA) are the most-used techniques to identify ATS using oral fluid [14]. Classical ELISA assays are laboratory tests that are based on the rivalry between the drug present in the sample and the same drug tagged with the enzyme (added to the system), for points of attachment of antibodies immobilized in the wells of a plate. Urine toxicology assessments deliver sensitive and accurate measures of various types of drugs; however, certain drugs such as ATS are confined or constrained to a limited timeline because of the fast metabolizing. The identification window can also be dependent on elements such as purity, the usage frequency, the person’s physical state (e.g., hydration degree), and the way the drug was ingested (e.g., oral, intranasal, intravenous injections) [15].

Macrocyclic compounds are important structures and key building blocks in supramolecular chemistry [16,17,18]. Among them, cucurbiturils are a relatively novel family of barrel-shaped macrocyclic hosts that can encapsulate guest molecules in their unique large cavities, which are edged by carbonyl oxygen atoms [19,20,21]. Cucurbiturils can be synthesized in a variety of different morphology sizes, with either 5, 6, 7, 8 or 10 glycoluril subunits [21,22,23,24]. In recent years, cucurbit[n]urils (CB[n]) were used as drug delivery vehicles for neutral molecules and cations due to their high affinity to form host–guest inclusion complexes [25,26,27]. Among the numerous cucurbiturils of different sizes, CB[7] which comprises seven glycoluril units is highly explored in the literature as a successful host molecule [28,29] with decreased toxicity and increased chemical and thermal stability [30,31]. 

In their study about the development of new point-of-use, portable and wireless drug sensor, Jang et al. showed the high sensitivity of the host molecule CB[7] (combined with organic field-effect transistors) toward ATS, thus providing a viable methodology for the fabrication of selective and sensitive drug sensors [32]. The potential of functionalized CB[7] was reported in some studies as a drug biosensor for the recognition of amphetamine and methamphetamine stimulants [32,33]. It is possible to better detail and quantify this type of interaction using quantum chemistry based on DFT study [34,35,36,37]. Indeed, atomistic calculations are necessary in providing answers on the nature and effect of inter-molecular interactions [38,39,40,41,42].

Herein, we present a theoretical study on the structural, electronic, biosensing and energetic properties of the inclusion of amphetamine and methamphetamine inside the cavity of CB[7]. The present work aims to investigate the stability of the formed complexes and to examine the nature of the intermolecular interactions involved in the complexation process using the new DFT-D4 dispersion correction model. 

## 2. Computational Procedures

The initial molecular structure of CB[7] was taken from the crystal structure determined by X-ray diffraction methods (CCDC 1542694) [32]. Both S and R enantiomers of amphetamine (AMP) and methamphetamine (MET) ((S)-amphetamine CID: 5826; (R)-amphetamine CID: 32893; (S)-methamphetamine CID: 10836 (R)-methamphetamine CID: 36604) were investigated in this study and their chemical 3D structures were retrieved from PubChem database server [43]. Then, protonated forms of AMP and MET were considered and built with Avogadro program [44] assuming that their amino groups are protonated as observed in the solid-state crystal structures of 1:1 inclusion complexes of AMP and MET with CB[7]. The geometries of all initial structures were subjected to prior geometry optimization with ORCA program (version 4.2.0) [45,46] in gas phase using BLYP functional [47,48] in conjunction with the atom-pairwise dispersion correction based on tight-binding partial charges (D4) [49,50] and def2-SVP basis set [51,52] without any symmetry constraints. A geometrical counterpoise correction (gCP) to the def2-SVP for the intra- and inter-molecular basis set superposition error was used [53]. Then, the computational process followed to calculate the most stable configurations (configurations of minimum energy) corresponding to the inclusion complexes of AMP and MET with CB[7] was based on the method developed by Liu and Guo [54]. In addition to Liu and Guo method, other techniques have been developed recently for the theoretical modelling of host–guest binding affinity [55,56,57,58,59,60]. Liu and Guo proposed the semi-empirical methods such as AM1 and PM3, which are fast and could be applied for several positions along the z-axis. We have extended the use of this method to examine accurately the non-covalent interactions (electrostatic interactions, van der Waals interactions, hydrogen bonds…) by applying exclusively DFT approach associated with the most recent dispersion correction functionals (BLYP-D4).

**Figure 1:**
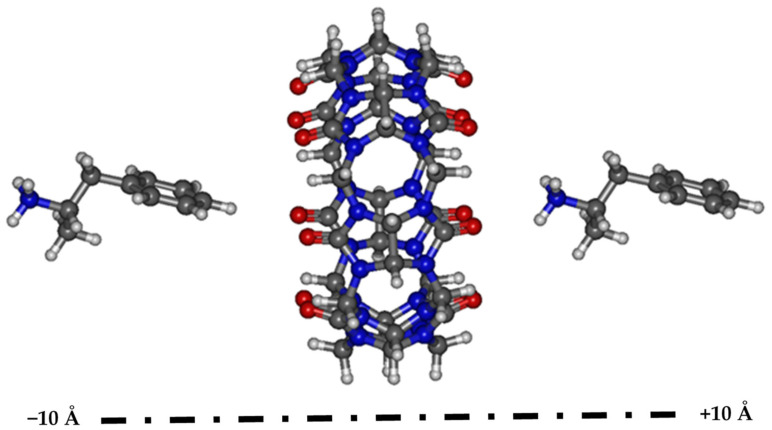
Docking orientations of S-AMP@CB[7] from −10 Å to +10 Å. Color code: carbon, grey; nitrogen, blue; oxygen, red; hydrogen, white.

The center of AMP, MET and CB[7] was set as the center of the coordination system, then AMP and MET guests, as well as their enantiomers, were translated along the Z-axis from −10 to +10 Å with 2 Å step as shown in Figure 1 using Gabedit program [61]. Each generated structure in this process was fully optimized at BLYP-D4/def2-SVP-gCP level of theory. Frequency computations were performed on AMP, MET, CB[7] and the most stable inclusion complexes to ensure that the optimized structures are minima on the potential energy surface. The most stable configuration for each complex (R-AMP@CB[7], S-AMP@CB[7], R-MET@CB[7] and S-MET@CB[7]) was further reoptimized at BLYP-D4/def2-TZVP-gCP in gas phase and aqueous phase. The water solvent effects were considered by applying the conductor-like polarizable continuum model (CPCM) [62,63]. The following Equation (1) was used for the calculation of complexation energies:

ΔE_Complexation_ = E_Complex_ − (E_free guest_ + E_free CB[7]_) (1)

where ∆E_complexation_ represents the complexation energy, E_complex_, E_free guest_ and E_free_ CB[7] are respectively the optimized energies of the complex, the free amphetamine or methamphetamine and the free cucurbit[7]uril.

The aqueous phase geometries were used to characterize the nature of the interactions occurring between the guest molecules (AMP and MET) and CB[7] by performing an independent gradient model (IGM) analysis based on Hirshfeld partition of electron density [64]. The non-covalent interactions (NCI) [65,66] maps were generated with Multiwfn software [67] and visualized using the Visual Molecular Dynamics (VMD) program [68]. To quantify the amount of charge transfer between CB[7] and AMP and MET, the charge decomposition analysis (CDA) [69,70] and extended charge decomposition analysis (ECDA) [71] were performed using the Multiwfn program [67]. 

## 3. Results and Discussions

### 3.1. Searching for the Most Stable Complexes

The complexation energies calculated in gas phase as a function of Z coordinate are reported in Table 1. The optimized structures show negative energies of formation, indicating therefore a favorable thermodynamic process. The most stable configurations for R-AMP@CB[7], S-AMP@CB[7], R-MET@CB[7] and S-MET@CB[7] with respective energies of −337.43, −346.79, −335.39 and −336.52 kJ/mol are located respectively at Z = 0 Å, Z = −6 Å, Z = −6 Å and Z = −4 Å (Table 1).

The S-enantiomer complexes (S-AMP@CB[7] and S-MET@CB[7]) are respectively slightly more stable by −9.36 and −1.13 kJ/mol in the gas phase than their corresponding R-enantiomers (R-AMP@CB[7] and R-MET@CB[7]).

For higher accuracy, we reoptimized each of the four most stable complexes at BLYP-D4/def2-TZVP-gCP level of theory in gas and aqueous phases. The calculated complexation energies are reported in Table 2. 

In gas phase, we note that as the basis set improves from def2-SVP to def2-TZVP, the computed complexation energies of R-AMP@CB[7], S-AMP@CB[7], R-MET@CB[7] and S-MET@CB[7] are −338.85, −349.58, −334.01 and −334.53 kJ/mol, respectively. The improvement of basis set quality does not lead to significant variation of the complexation energies (Table 2).

In aqueous phase, a significant decrease in the complexation energy values was noted (Table 2); however, the results show a similar trend with those obtained in gas phase calculations. Indeed, S-AMP@CB[7] and S-MET@CB[7] are found to be 3.18 and 1.31 kJ/mol more stable than the complexes R-AMP@CB[7] and R-MET@CB[7]. The small difference in their complexation energies suggests the formation of a racemic mixture of AMP and MET enantiomers [72], as observed in the X-ray crystal structures of the host–guest complexes where both forms of R and S enantiomers were found. Based on the frequency calculations, we determined ΔG_Complexation_ at temperature 298K for each complex and compared it with the experimental values. The experimental ΔG_Complexation_ values of AMP@CB[7] and MET@CB[7] are −34.7 and −33.8 kJ/mol, which are very close to the computed values (−45.5 and −35.3 kJ/mol, respectively). Therefore, our calculations results are in good agreement with the experimental data.

It is also clear that complexes of R-AMP@CB[7] and S-AMP@CB[7] are more stable than their analogs R-MET@CB[7] and S-MET@CB[7] in gas phase while in water solvent the stability is reversed, and the latter complexes become more stable. The water solvent significantly decreased the complexation energy, most likely due to the large dipole moments of the complexes in water. Indeed, the calculated dipole moments of R-AMP@CB[7], S-AMP@CB[7], R-MET@CB[7] and S-MET@CB[7] in water are 18.03, 14.93, 17.32 and 17.88 D, respectively, and are larger than their corresponding values of 9.23, 8.73, 9.26 and 9.19 D in gas phase.

Further, single-point energy calculations were performed on BLYP-D4/def2-TZVP optimized geometries at B3LYP-D4/def2-TZVP level of theory [73], both functionals show the same behavior for the evaluation of the complexation energies as shown in Table S1 (Supplementary Data).

Using a simple thermodynamic study, it is possible to determine which guest interacts most strongly with CB[7]. The idea will be based on the computation of standard free energy change ΔΔG*_Sol_ for the following guest exchange reaction in solution (2):

(2) MET@CB[7](aq)+AMP (aq)→AMP@CB[7](aq)+MET (aq)

**Scheme 1:**
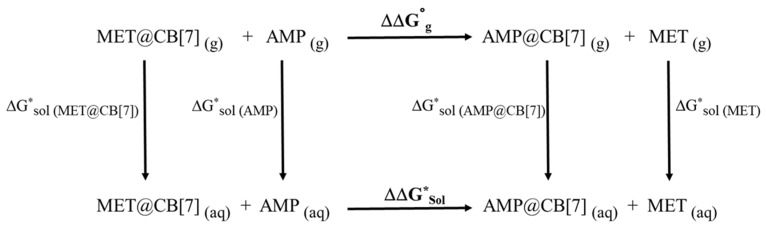
Thermodynamic cycle used to calculate the free energy of reaction 1 from its components.

As shown in Scheme 1, ΔΔG*_Sol_ can be determined from its components by introducing a thermodynamic cycle using Equation (3):

(3) $$\Delta \Delta G_{\mathrm{sol}}^{*}=\Delta \Delta G_{g}^{0}+\Delta G_{\mathrm{sol}\left(\mathrm{AMP}@\mathrm{CB}\left[7\right]\right)}^{*}+\Delta G_{\mathrm{sol}\left(\mathrm{MET}\right)}^{*}-\Delta G_{\mathrm{sol}\left(\mathrm{MET}@\mathrm{CB}\left[7\right]\right)}^{*}-\Delta G_{\mathrm{sol}\left(\mathrm{AMP}\right)}^{*}$$

where ΔΔG^0^_g_ is the change of free energy in the gas-phase reaction; ΔG*_sol(AMP@CB[7])_, ΔG*_sol(MET)_, ΔG*_sol(MET@CB[7])_ and ΔG*_sol(AMP)_ are, respectively, the solvation free energies of AMP@CB[7], (MET), MET@CB[7] and (AMP) species in water.

MET and AMP species exhibit several isomers, their Gibbs free energies have been evaluated using a Boltzmann distribution according to the following relation (4):

(4) $$G_{\left[A\right]}^{0}=-RT\ln\sum_{i\in [A]}e^{-G_{i}^{0}/RT}$$

where [*A*] emphasizes calculation over the population of the two enantiomers of each guest. Gas phase free energies of all species were estimated at *T* = 298.15 K and *p* = 1 atm through the computation of their energy and geometry at BLYP-D4/def2-SVP-gCP level of theory. Gibbs free energies of aqueous solvation were computed for all species using the conductor-like polarizable continuum model (CPCM) using BLYP-D4/def2-TZVP-gCP. Table 3 reports calculated values of all thermodynamic parameters used in the current study. From this Table, we have noticed that the standard free energy change ΔΔG*_sol_ is 12.7 kJ/mol. The positive value of ΔΔG*_sol_ indicates that the exchange reaction moves in the direction of the formation of MET@CB[7] complex, therefore, CB[7] forms complex with methamphetamine more favorably than with amphetamine. 

### 3.2. Geometries of the Most Stable Complexes of AMP and MET with CB[7]

**Figure 2:**
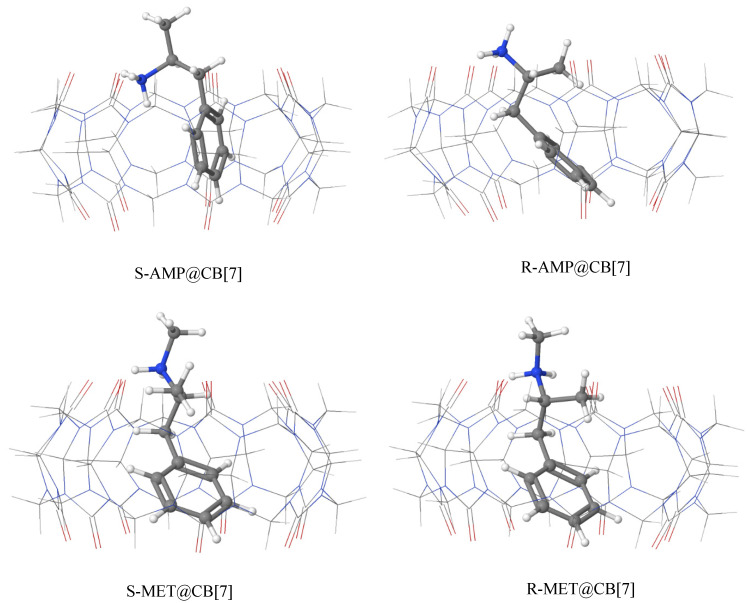
Structures of the most stable conformations optimized at BLYP-D4-def2-TZVP-gCP level in aqueous phase.

The geometries of fully optimized structures of all inclusion complexes calculated at BLYP-D4-def2-TZVP-gCP level in water solvent (Supplementary Table S2) are visualized using Jmol viewer [74] and illustrated in Figure 2. 

DFT simulations show that AMP and MET are encapsulated inside CB[7]. Indeed, the benzyl moiety of AMP and MET is deeply inserted into CB[7] due to hydrophobic interactions between the CB[7] cavity and the phenyl ring, whereas the ammonium group of AMP and the methylammonium group of MET are found outside the cavity, at the top of CB[7] leaning to the closest carbonyl groups at the portals, in agreement with the experimental structures. The calculated shortest N…O bond lengths for AMP@CB[7] and MET@CB[7] are both equal to 2.85 Å and are close to the experimental bond values of 2.76 and 2.78 Å, respectively, involving effective ion–dipole interactions between CB[7] and the ammonium groups. 

### 3.3. Analysis of Non-Covalent Interactions

**Figure 3:**
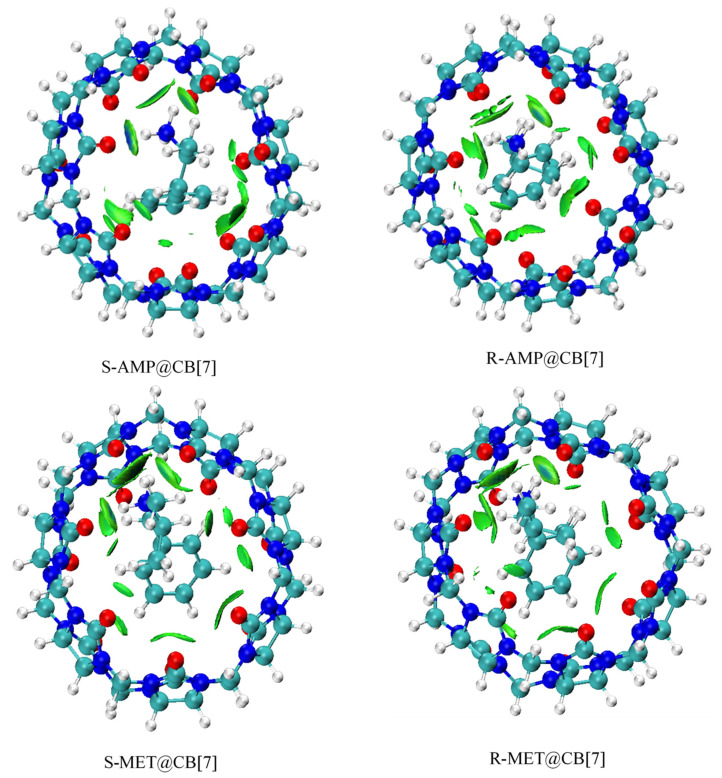
IGM isosurfaces plots (isovalue = 0.005 a.u.) of the studied complexes.

The characterization of non-covalent interactions (NCI) is of great importance in molecular recognition systems. We have employed NCI analysis to understand the nature of intermolecular interactions occurring in AMP@CB[7] and MET@CB[7] complexes through the isosurface map of the independent gradient model (IGM) approach. IGM Plots for all compounds were generated with an isovalue of 0.005 a.u. and shown in Figure 3, where blue and green colors represent respectively strong attractive interactions and weak van der Waals interactions.

As observed in Figure 3, the IGM isosurfaces indicate the presence of weak van der Waals interactions (green colored fields) and hydrogen bonds (blue disks) between the hydrogen atoms of the ammonium groups of AMP and MET and the lone pairs on the oxygen atoms of CB[7]. The calculated hydrogen bonds (N—H···O) of the studied complexes (Supplementary Figure S1) are within the range 1.87–2.72 Å. Both weak van der Waals interactions and intermolecular hydrogen bonding [34] stabilize the formation of AMP@CB[7] and MET@CB[7] complexes. 

### 3.4. Biosensing Properties 

The real-time application of drug biosensing based on organic field-effect transistors sensors is a promising platform for detecting illegal drugs such as amphetamine and methamphetamine. Jang et al. showed that OFET based sensors functionalized with CB[7] derivatives for the binding of amphetamine and methamphetamine exhibit highly sensitive and selective sensing properties in water, urine and physiological buffers [32]. Analysis of frontier molecular orbitals (the HOMO and LUMO) is important for the computational design of drug sensors. 

According to the energies of HOMO and LUMO levels reported in Table 4, a decrease of the HOMO–LUMO gaps is observed upon the formation of the inclusion complexes, due to the lowering of the LUMO levels, whereas the energies of the HOMO levels remain almost unchanged, suggesting a charge transfer between CB[7] and AMP and MET, therefore, the variation of electric conductivity can be influenced owing to the charges transfer between AMP and MET and CB[7]. These results indicate that miniaturized electrical biosensors devices can be used to detect AMP and MET in urine or biological fluid samples. 

**Figure 4:**
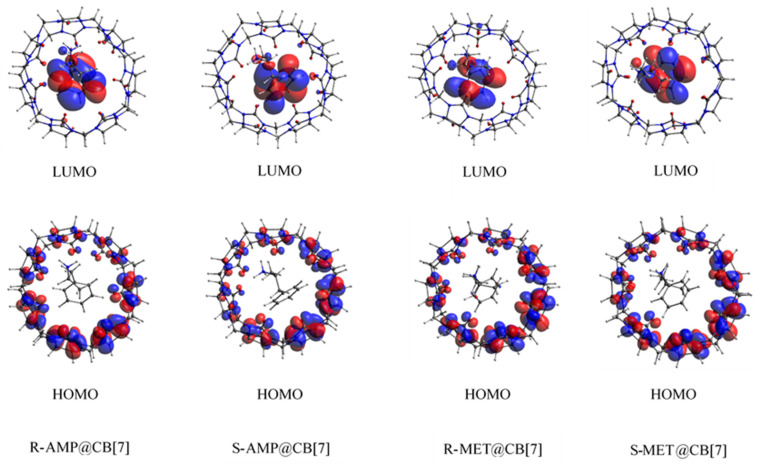
HOMO and LUMO plots of R-AMP@CB[7], S-AMP@CB[7], R-MET@CB[7] and S-AMP@CB[7].

Frontier molecular orbital (HOMO and LUMO) plots for the studied complexes are shown in Figure 4. The analysis of HOMO and LUMO plots reveals that the HOMOs are almost exclusively spread over the CB[7] while the LUMOs are mainly localized on AMP and MET and almost entirely on their aromatic ring moieties.

### 3.5. Charge Decomposition and Extended Charge Decomposition Analysis

The CDA and ECDA analysis methods can provide insight into the charge transfer (donation and back donation) between fragments in non-covalent complexes. The calculated results indicate that the CDA and ECDA analysis of R-AMP@CB[7], S-AMP@CB[7], R-MET@CB[7] and S-AMP@CB[7] follow the same trend as shown in Table 4. An electron donation (d) from CB[7] to guest (AMP or MET) in the range [0.21–0.23] was obtained from CDA analysis, while the back-donation (b) occurring from the guests (AMP or MET) to the CB[7] is in the range [0.042−0.071]. The ECDA analysis confirms the net charge transfer obtained by AMP or MET from CB[7] with an approximate value of 0.2 (Table 5), indicating that intermolecular charge transfer is involved in stabilizing the formed complexes.

## 4. Conclusions

DFT calculations including D4 dispersion correction were carried out to investigate the inclusion process between CB[7] and amphetamine-type stimulant drugs. The conclusions are as followed:

According to the computed complexation energies, the inclusion process of amphetamine and methamphetamine drugs in CB[7] is favorable in gas phase and in water solution.

In gas phase, R-AMP@CB[7] and S-AMP@CB[7] are thermodynamically more favored than R-MET@CB[7] and S-MET@CB[7], while the latter are more favored than the former in water solvent.

The presence of a mixture of R and S enantiomers was evidenced by DFT-D4 calculations, with a small energy difference in favor of the S enantiomers.

X-ray data support the theoretically predicted inclusion conformations of amphetamine and methamphetamine in the CB[7] with the phenyl ring moiety deeply inserted inside the CB[7] cavity and the ammonium group outside the cavity.

Structural analysis indicated that van der Waals forces, intermolecular hydrogen bonds, charge transfer interactions and ion-dipole interactions between CB[7] and amphetamine and methamphetamine play a major role in the process of complex stabilization. 

Frontier molecular orbital analysis of all complexes revealed that the LUMO is shifted to lower energy and stabilized upon complexation, the resulting decrease in the HOMO-LUMO gap leads to a variation of the conductivity, making them suitable for biosensing applications. 

These theoretical results highlight the high affinity of cucurbiturils for cationic guests through cooperative non-covalent interactions and their potential as stabilizing hosts and a good sensing candidates for the detection of drugs in real samples for practical applications. 

## Figures and Tables

**Table 1:** Calculated complexation energies (in kJ/mol) of CB[7] with R-AMP, S-AMP, R-MET and S-MET at BLYP-D4/def2-SVP-gCP level.

Docking Configurations	R-AMP@CB[7]	S-AMP@CB[7]	R-MET@CB[7]	S-MET@CB[7]
−10 Å	−301.08	−323.76	−334.75	−284.33
−8 Å	−299.69	−295.34	−269.57	−331.66
−6 Å	−324.09	**−346.79**	**−335.39**	−320.60
−4 Å	−324.33	−346.60	−334.80	**−336.52**
−2 Å	−337.34	−337.14	−334.73	−317.27
0 Å	**−337.43**	−337.02	−334.71	−317.46
+2 Å	−320.76	−337.54	−334.64	−322.59
+4 Å	−300.51	−300.90	−282.05	−276.98
+6 Å	−300.58	−300.91	−277.80	−291.80
+8 Å	−300.60	−289.05	−278.07	−287.32
+10 Å	−300.71	−295.21	−278.39	−286.65

**Table 2:** Calculated complexation energies and standard Gibbs free energy (in kJ/mol) of CB[7] with AMP and MET at BLYP-D4/def2-TZVP-gCP level.

Complex	ΔE_Complexation_ (kJ/mol)		ΔG_Complexation_ (kJ/mol) ^(a)^	
	Gas Phase	Aqueous Phase	Experimental	Calculated
R-AMP@CB[7]	−338.85	−73.23	−34.7	−45.5
S-AMP@CB[7]	−349.58	−76.41		
R-MET@CB[7]	−334.01	−84.01	−33.8	−35.3
S-MET@CB[7]	−334.53	−85.32		

^(a)^ ΔG_Complexation_ values are calculated in aqueous phase at 298 k.

**Table 3:** Thermodynamic parameters used for the computation of standard free energy change.

Species	ΔG^0^_gas_ (a.u.)	ΔG*_sol_ (a.u.)	ΔΔG^0^_gas_ (kJ/mol)	ΔΔG*_sol_ (kJ/mol)
AMP	−405.16178	−405.26800	−10.2	12.7
MET	−444.38724	−444.48477		
AMP@CB[7]	−4612.20227	−4612.38314		
MET@CB[7]	−4651.42384	−4651.604737		

**Table 4:** Calculated HOMO and LUMO energy levels (eV) and HOMO–LUMO energy gaps (eV) of CB[7], R-AMP@CB[7], S-AMP@CB[7], R-MET@CB[7] and S-MET@CB[7].

System	E_HOMO_ (eV)	E_LUMO_ (eV)	∆E_LUMO-HOMO_ (eV)
CB[7]	−5.62	−0.52	5.10
R-AMP@CB[7]	−5.68	−0.95	4.73
S-AMP@CB[7]	−5.68	−1.13	4.55
R-MET@CB[7]	−5.67	−0.95	4.72
S-MET@CB[7]	−5.68	−0.98	4.70

**Table 5:** Charge decomposition (CDA) analysis and extend charge decomposition analysis (ECDA) of CB[7], R-AMP@CB[7], S-AMP@CB[7], R-MET@CB[7] and S-MET@CB[7].

CDA					ECDA
System	d	b	d-b	r	Net Electrons Obtained by AMP or MET
R-AMP@CB[7]	0.232	0.071	0.161	−0.320	0.196
S-AMP@CB[7]	0.209	0.042	0.167	−0.306	0.206
R-MET@CB[7]	0.232	0.069	0.163	−0.323	0.199
S-MET@CB[7]	0.231	0.065	0.166	−0.335	0.203

d: number of electrons donated from CB[7] to guest (AMP or MET); b: number of electrons back-donated from the guest (AMP or MET) to CB[7]; r: number of electrons involved in repulsive polarization.

## Data Availability

The data presented in this study are available on request from the corresponding author.

## Supplementary Materials

The following are available online. The intermolecular hydrogen bonds between the hydrogen atoms of the ammonium groups of AMP and MET and the oxygen atoms of CB[7] are shown in Supplementary Figure S1. Table S1 represents the B3LYP-D4/ def2-TZVP single-point calculations (in italic) on BLYP-D4/def2-TZVP (in bold) optimized structure. The atomic coordinates of optimized structures of S-AMP@CB[7], R-AMP@CB[7], S-MET@CB[7] and R-MET@CB[7] obtained at BLYP-D4-def2-TZVP-gCP level of theory in water solvent are listed in Supplementary Table S2.

## Sample Availability

No samples are available Samples of the compounds are not available from the authors.
