# Supplementary material for: DFT-D4 Insight into the Inclusion of Amphetamine and Methamphetamine in Cucurbit[7]uril: Energetic, Structural and Biosensing Properties
Source: Molecules. 2021 Dec 10;26(24):7479. doi: 10.3390/molecules26247479 (PMC8705717; doi:10.3390/molecules26247479)
Supplement: Supplementary file 1 [file molecules-26-07479-s001.zip › molecules-1485321-supplementary.pdf]

DFT-D4 Insight into the Inclusion of Amphetamine and Methamphetamine in Cucurbit[7]uril: Energetic, Structural and Biosensing Properties

Abdelkarim Litim [1], Youghourta Belhocine [2], Tahar Benlecheb [1,*], Monira Galal Ghoniem [3], Zoubir Kabouche [1], Fatima Adam Mohamed Ali [3,*], Babiker Yagoub Abdulkhair [4,5], Mahamadou Seydou [6] and Seyfeddine Rahali [7,*]

[1] Laboratory of Sensors, Instrumentations and Process (LCIP), University of Abbes Laghrour, 40000 Khenchela, Algeria; Litim.abdelkarim@univ-khenchela.dz (A.L.); Kabouche.zoubir@univ-khenchela.dz (Z.K.)

[2] Department of Petrochemical and Process Engineering, Faculty of Technology, 20 August 1955 University of Skikda, P.O. Box 26, El Hadaik Road, 21000 Skikda, Algeria; y.belhocine@univ-skikda.dz

[3] Department of Chemistry, College of Science, Imam Mohammad Ibn Saud Islamic University (IMSIU), Riyadh 11432, Saudi Arabia; mgghoniem@imamu.edu.sa

[4] Chemistry Department, College of Science, Imam Mohammad Ibn Saud Islamic University (IMSIU), Riyadh 11432, Saudi Arabia; byabdulkhair@imamu.edu.sa

[5] Chemistry Department, Sudan University of Science and Technology (SUST), 13311 Khartoum, Sudan

[6] Université de Paris, CNRS, ITODYS, F-75013 Paris, France; mahamadou.seydou@univ-paris-diderot.fr

[7] Department of Chemistry, College of Science and Arts, Qassim University, Ar Rass, Saudi Arabia

* Correspondence: Famohamedali@imamu.edu.sa (F.A.M.A.); benlecheb.tahar@univ-khenchela.dz (T.B.); s.rahali@qu.edu.sa (S.R.)

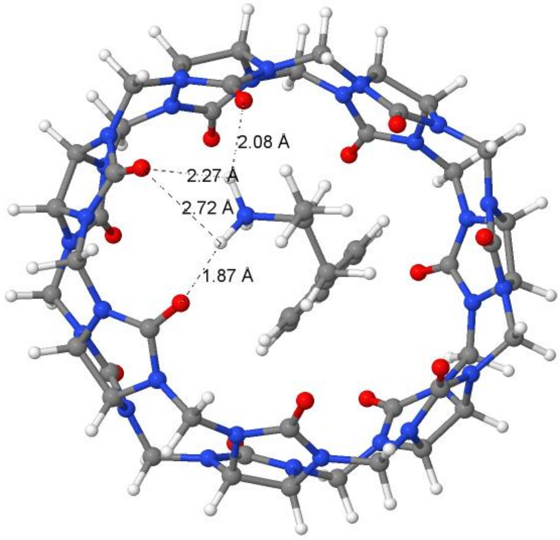

S-AMP@CB[7]

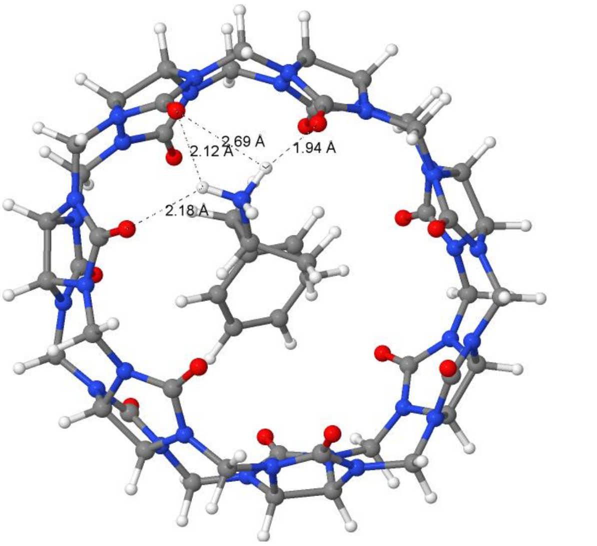

R-AMP@CB[7]

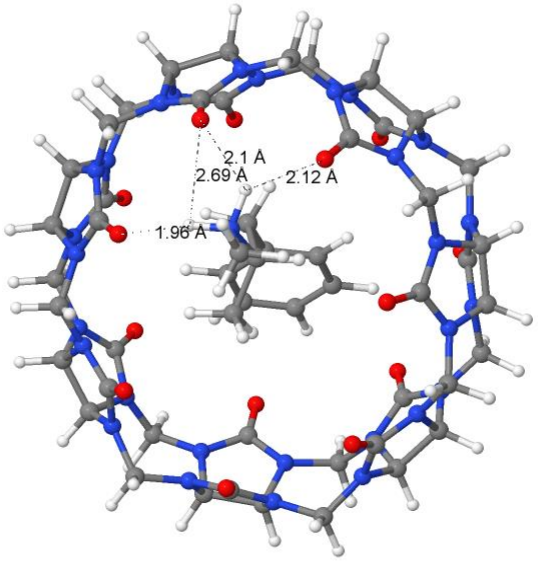

S-MET@CB[7]

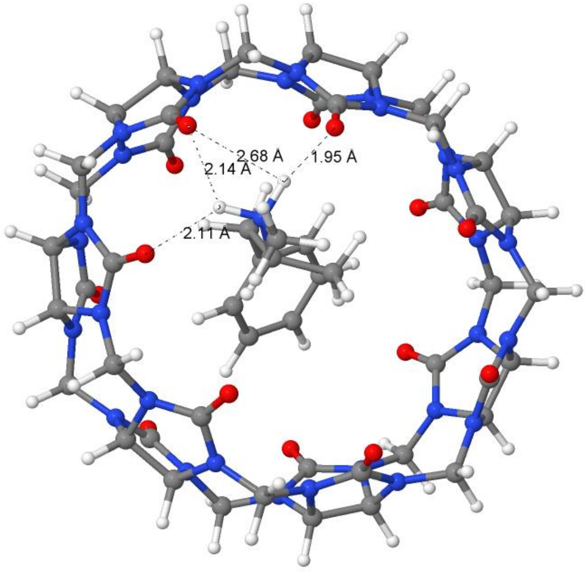

R-MET@CB[7]

Fig. S1 Intermolecular hydrogen bonds between the hydrogen atoms of the ammonium groups of AMP and MET and the oxygen atoms of CB[7]

Table S1 B3LYP-D4/ def2-TZVP single-point calculations (in italic) on BLYP-D4/def2-TZVP (in blod) optimized structure

**Complex**	**Calculation methods**	**Complexation energy (kJ/mol)**
R-AMP@CB7 (gas phase)	BLYP-D4	**-338.85**
	B3LYP-D4	*-348.01*
S-AMP@CB7 (gas phase)	BLYP-D4	**-349.58**
	B3LYP-D4	-*358.85*
R-AMP@CB7 (aqueous phase)	BLYP-D4	**-73.23**
	B3LYP-D4	*-74.43*
S-AMP@CB7 (aqueous phase)	BLYP-D4	**-76.41**
	B3LYP-D4	*-76.17*
R-MET@CB7 (gas phase)	BLYP-D4	**-334.01**
	B3LYP-D4	-*343.84*
S-MET@CB7 (gas phase)	BLYP-D4	**-334.53**
	B3LYP-D4	-*344.25*
R-MET@CB7 (aqueous phase)	BLYP-D4	**-84.01**
	B3LYP-D4	*-72.64*
S-MET@CB7 (aqueous phase)	BLYP-D4	**-85.32**
	B3LYP-D4	-*85.55*

Table S2 Cartesian coordinates (in angstroms) of optimized structures of S-AMP@CB[7], R-AMP@CB[7], S-MET@CB[7] and R-MET@CB[7] obtained at BLYP-D4/def2-TZVP-gCP level of theory in aqueous solution.

- S-AMP@CB[7]

XYZ file generated by gabedit : coordinates in Angstrom

O	7.3661330000	6.8972250000	14.7027550000
O	6.2518430000	3.3096930000	14.2171200000
O	8.0758830000	0.0387460000	14.4239960000
O	11.6139230000	-0.4699150000	15.1172940000
O	14.0913790000	2.2331880000	15.7056440000
O	13.5274330000	6.0059020000	15.8875400000
O	10.6685880000	8.2543710000	15.3531530000
O	5.9749210000	6.1885370000	20.6848690000
O	4.6361620000	2.8651970000	20.1675140000
O	6.5525760000	-0.1830070000	20.4037640000
O	10.1145130000	-0.2395820000	21.0898470000
O	12.8761210000	2.5181740000	21.6121120000
O	12.9618590000	5.7591420000	21.9652240000
O	9.7602250000	7.0842820000	21.2096050000
N	7.0981650000	8.1337310000	16.6649480000
N	5.6328910000	6.4922440000	16.2182490000
N	4.8288390000	4.2205810000	15.8299860000
N	5.0455400000	1.9917020000	15.7239660000
N	6.2738130000	-0.1118590000	15.9045370000
N	8.0626450000	-1.4206800000	16.2458660000
N	10.4360860000	-1.5698480000	16.8118740000
N	12.5135090000	-0.8314580000	17.2433230000
N	14.2574690000	0.8559690000	17.5866640000
N	14.9144340000	2.9937830000	17.7592410000
N	14.6484430000	5.4214040000	17.8561000000
N	13.5996160000	7.4089570000	17.7521640000
N	11.5246670000	8.6828070000	17.4871140000
N	9.3369510000	9.0113080000	17.1177900000
N	6.6652650000	7.7072810000	19.0483430000
N	4.9554570000	6.3343510000	18.5895960000
N	4.1932700000	4.0464870000	18.2004260000
N	4.3995120000	1.8157120000	18.0930210000
N	5.6055570000	-0.2983120000	18.2736570000
N	7.5264300000	-1.3883390000	18.6541120000
N	9.8905130000	-1.6137330000	19.2130600000
N	11.8785900000	-0.6572090000	19.6132080000
N	13.5987130000	1.0420550000	19.9482130000
N	14.6763450000	2.9919250000	20.2014970000
N	14.5862430000	5.4337790000	20.3198940000
N	13.2683860000	7.2336090000	20.1837800000
N	11.1573830000	8.4140230000	19.8949900000
N	8.9546300000	8.4328160000	19.4767760000
C	6.7670030000	7.1414700000	15.7515290000
C	4.8632400000	5.5939980000	15.3675600000
H	5.3265710000	5.6016250000	14.3769810000
H	3.8312500000	5.9699220000	15.2974510000
C	5.4722650000	3.1870900000	15.1623850000
C	5.3399700000	0.6972100000	15.1361360000

H	5.7873790000	0.8708010000	14.1532900000
H	4.3986010000	0.1397020000	15.0217520000
C	7.5315460000	-0.4384580000	15.4208360000
C	9.3379840000	-2.0634890000	15.9893020000
H	9.2249700000	-3.1434380000	16.1617780000
H	9.6015280000	-1.8810290000	14.9433960000
C	11.5277090000	-0.9013320000	16.2671660000
C	13.8739640000	-0.3996550000	16.9631300000
H	13.9651560000	-0.2762490000	15.8798350000
H	14.5688160000	-1.1803170000	17.3058390000
C	14.3786610000	2.0505290000	16.8886860000
C	15.3897880000	4.2908970000	17.3074310000
H	16.4510780000	4.4020630000	17.5780760000
H	15.2845450000	4.3172830000	16.2182200000
C	13.8779510000	6.2491100000	17.0419320000
C	12.9380210000	8.5610480000	17.1591500000
H	13.0136210000	8.4728610000	16.0711880000
H	13.4637320000	9.4664890000	17.4942590000
C	10.5234930000	8.6002720000	16.5258210000
C	8.0906190000	9.1600080000	16.3873260000
H	7.6553410000	10.1402690000	16.6286290000
H	8.3287760000	9.1156400000	15.3207600000
C	6.1566600000	8.2057160000	17.7689850000
H	5.7703290000	9.2278660000	17.8800860000
C	5.0617710000	7.1538590000	17.3931180000
H	4.0870840000	7.5960140000	17.1451100000
C	3.8680410000	3.7411310000	16.8133730000
H	2.8590320000	4.1033460000	16.5691420000
C	4.0165900000	2.1853510000	16.7375180000
H	3.0923990000	1.6655230000	16.4469400000
C	5.8623040000	-0.9744030000	17.0117190000
H	4.9942490000	-1.5826350000	16.7205890000
C	7.1502610000	-1.8164670000	17.3071050000
H	6.9850010000	-2.9028550000	17.2851120000
C	10.7278680000	-2.1161770000	18.1360250000
H	10.7010790000	-3.2145750000	18.1098620000
C	12.1380290000	-1.5251390000	18.4655950000
H	12.8889660000	-2.2847230000	18.7231720000
C	14.6900670000	0.9718330000	18.9667930000
H	15.3878840000	0.1648830000	19.2274340000
C	15.3138970000	2.4026980000	19.0371380000
H	16.4066120000	2.4126900000	19.1480740000
C	15.0393040000	6.0873270000	19.0997590000
H	16.1255290000	6.2442890000	19.1291770000
C	14.2057530000	7.4143860000	19.0721120000
H	14.8055990000	8.3219990000	19.2221080000
C	11.0476500000	9.2805130000	18.7317350000
H	11.5436530000	10.2418000000	18.9205960000
C	9.5044540000	9.3910980000	18.5083820000
H	9.0952490000	10.3927360000	18.6936960000
C	5.8778980000	6.6817500000	19.5599590000
C	3.9214800000	5.3369730000	18.8075910000
H	3.8331970000	5.1833560000	19.8869820000
H	2.9738670000	5.7265120000	18.4094440000
C	4.4439350000	2.9053550000	18.9525560000

C	4.3929620000	0.4433190000	18.5678040000
H	4.2780010000	0.4694790000	19.6556390000
H	3.5408500000	-0.0846780000	18.1159820000
C	6.5640360000	-0.5712610000	19.2355790000
C	8.5330720000	-2.0716230000	19.4498180000
H	8.2975250000	-1.8956800000	20.5039470000
H	8.4893490000	-3.1497430000	19.2362100000
C	10.5731320000	-0.7733450000	20.0776420000
C	12.9432980000	-0.1544170000	20.4600960000
H	12.5073120000	0.0934340000	21.4318370000
H	13.7039540000	-0.9408580000	20.5884050000
C	13.6349490000	2.2176130000	20.6803250000
C	15.1447870000	4.2052000000	20.8521210000
H	16.2374520000	4.2407950000	20.7540740000
H	14.8746520000	4.1532680000	21.9109510000
C	13.5428580000	6.1027260000	20.9294000000
C	12.3789100000	8.2719930000	20.6687330000
H	12.1083170000	8.0173690000	21.6974200000
H	12.9011850000	9.2384840000	20.6546660000
C	9.9413580000	7.8920720000	20.2834240000
C	7.5559000000	8.4771920000	19.8937550000
H	7.2366920000	9.5305630000	19.9054720000
H	7.4896710000	8.0621910000	20.9035260000
N	10.5872530000	4.3096400000	21.0940140000
C	9.4946790000	3.3849400000	21.6327210000
C	8.2067300000	3.6210950000	20.8168570000
C	8.4108970000	3.4125750000	19.3279240000
C	9.3294800000	3.6343200000	23.1301610000
C	8.6598730000	2.1220760000	18.8269890000
C	8.3975200000	4.4920550000	18.4283870000
C	8.8782330000	1.9156490000	17.4636440000
C	8.6011940000	4.2846200000	17.0573500000
C	8.8374900000	2.9944760000	16.5711630000
H	9.8730540000	2.3704220000	21.4535420000
H	7.8257370000	4.6323250000	21.0118660000
H	7.4515790000	2.9161300000	21.1896220000
H	8.5817740000	2.9378400000	23.5290530000
H	8.9820240000	4.6584780000	23.3263760000
H	10.2712160000	3.4709560000	23.6705310000
H	8.6744240000	1.2806650000	19.5163270000
H	8.2164100000	5.4987050000	18.7999880000
H	10.6476270000	4.1888080000	20.0732090000
H	11.5138590000	4.0795980000	21.4996050000
H	9.0818020000	0.9137620000	17.0986780000
H	8.5741980000	5.1269590000	16.3689310000
H	8.9787930000	2.8258730000	15.5053630000
H	10.3687440000	5.3165510000	21.2742090000

- R-AMP@CB[7]

XYZ file generated by gabedit : coordinates in Angstrom

O	13.7233760000	6.7902210000	16.1307240000
O	13.1103090000	3.3361210000	14.6404580000
O	12.1859510000	-0.4035750000	15.5151440000
O	11.5124470000	-1.8003830000	18.4071210000

O	11.6212220000	0.8107540000	20.9816640000
O	12.4401020000	4.7467490000	21.5560540000
O	13.3657760000	7.5396670000	19.4644120000
O	7.7409330000	7.6091810000	14.7165310000
O	7.4712610000	4.6147170000	12.5056120000
O	6.5507980000	1.4172760000	14.0440920000
O	5.8447000000	0.4857800000	17.7045870000
O	5.6410760000	2.2096550000	20.9799820000
O	6.3529530000	5.7544640000	21.3931730000
O	7.2295530000	8.0700470000	18.5917850000
N	12.1006790000	8.4675830000	16.0555260000
N	12.1467320000	6.9455670000	14.4085310000
N	11.9925070000	4.8215450000	13.2260940000
N	11.4829070000	2.6360120000	13.1160650000
N	10.8393780000	0.3790360000	13.7697820000
N	10.1243310000	-1.3623220000	14.9855570000
N	9.5499190000	-2.2007660000	17.2108990000
N	9.3947710000	-1.6579630000	19.3768700000
N	9.5470100000	-0.2046340000	21.3304610000
N	9.9189790000	1.8158730000	22.2338700000
N	10.3707110000	4.2022910000	22.5064210000
N	10.8740490000	6.3674980000	22.1611920000
N	11.3261930000	8.0732270000	20.4751380000
N	11.7675370000	8.8977930000	18.4373790000
N	9.6657800000	8.5636530000	15.6482840000
N	9.8388600000	7.4537590000	13.7034410000
N	9.6968350000	5.3307850000	12.4921540000
N	9.2636090000	3.1509790000	12.1888280000
N	8.5767560000	0.9419610000	12.9821700000
N	7.8811670000	-0.4179900000	14.6254090000
N	7.3596870000	-1.1066640000	16.9043610000
N	7.0606420000	-0.9121360000	19.1233760000
N	7.1879650000	0.4628430000	21.1341660000
N	7.5081530000	2.2989450000	22.3822800000
N	7.9588440000	4.6792910000	22.7041560000
N	8.4470440000	6.6946650000	21.8456870000
N	8.9018660000	8.4251910000	20.1808200000
N	9.3291470000	8.9418170000	18.0366520000
C	12.7568090000	7.3330940000	15.5983540000
C	12.7872100000	6.0025160000	13.5081930000
H	13.7181350000	5.6803500000	13.9827020000
H	13.0136810000	6.5018040000	12.5536710000
C	12.2800270000	3.5779460000	13.7640160000
C	11.8355900000	1.2258120000	13.1325750000
H	12.7661800000	1.1173770000	13.6965580000
H	11.9891970000	0.8853750000	12.0964330000
C	11.1576440000	-0.4542530000	14.8306090000
C	10.1653600000	-2.4730680000	15.9236010000
H	9.6567300000	-3.3278850000	15.4613900000
H	11.2128930000	-2.7261000000	16.1092260000
C	10.2801580000	-1.8734810000	18.3365260000
C	9.8426100000	-1.5053640000	20.7508840000
H	10.9272750000	-1.6478310000	20.7603380000
H	9.3664800000	-2.2717580000	21.3781510000
C	10.4783260000	0.8087450000	21.4626010000

C	10.7302590000	2.8415260000	22.8778340000
H	10.6401820000	2.7262570000	23.9705730000
H	11.7704120000	2.6822670000	22.5790180000
C	11.3459950000	5.0735250000	22.0128850000
C	11.6425200000	7.5426190000	21.7906150000
H	12.7009620000	7.2658850000	21.7836150000
H	11.4685760000	8.3182870000	22.5501210000
C	12.2694420000	8.0960240000	19.4533040000
C	12.5182930000	9.2058000000	17.2341050000
H	12.4217790000	10.2795080000	17.0212350000
H	13.5660320000	8.9599180000	17.4293300000
C	11.0214250000	8.8788370000	15.1803190000
H	11.1032100000	9.9497600000	14.9457560000
C	11.1837870000	7.9447960000	13.9391000000
H	11.5679650000	8.4558770000	13.0443100000
C	11.0034200000	4.7618780000	12.1631880000
H	11.3986560000	5.2191930000	11.2454580000
C	10.7068260000	3.2315120000	12.0288020000
H	11.0112590000	2.8040590000	11.0634330000
C	9.6217120000	-0.0593680000	13.0731660000
H	9.8672710000	-0.4588760000	12.0802300000
C	9.0343530000	-1.1192440000	14.0571030000
H	8.7235720000	-2.0555020000	13.5744540000
C	8.1160230000	-2.2288840000	17.4603070000
H	7.6886370000	-3.1847510000	17.1277300000
C	8.0151970000	-1.9960010000	19.0057510000
H	7.6768200000	-2.8777970000	19.5667060000
C	8.2970030000	0.0966310000	22.0115090000
H	8.0150980000	-0.7344940000	22.6723040000
C	8.6037440000	1.4297530000	22.7650750000
H	8.6532280000	1.3205020000	23.8573690000
C	9.2980390000	4.9391300000	23.1919730000
H	9.3553810000	4.7749720000	24.2775880000
C	9.5582650000	6.4196740000	22.7651090000
H	9.5458350000	7.1316720000	23.6026980000
C	10.2302930000	9.0115570000	20.2358400000
H	10.2584330000	9.8274380000	20.9728930000
C	10.4808010000	9.4842350000	18.7641160000
H	10.5177080000	10.5772970000	18.6507440000
C	8.9500140000	7.8414530000	14.6974900000
C	9.4335090000	6.7644190000	12.4910200000
H	8.3543520000	6.8964640000	12.3702810000
H	9.9588040000	7.2244970000	11.6413150000
C	8.6792760000	4.3885620000	12.4118950000
C	8.5000190000	1.9424250000	11.9297090000
H	7.4528950000	2.2364360000	11.8168090000
H	8.8614150000	1.4864810000	10.9978100000
C	7.5611870000	0.7294580000	13.9070480000
C	6.9301070000	-1.0645600000	15.5126200000
H	5.9988170000	-0.4927550000	15.4654400000
H	6.7506300000	-2.0941050000	15.1667940000
C	6.6722350000	-0.4036040000	17.8913560000
C	6.4322560000	-0.5190670000	20.3721910000
H	5.4612740000	-0.0723250000	20.1401680000
H	6.2886090000	-1.4215520000	20.9831920000

C	6.6698560000	1.7167150000	21.4386770000
C	7.1710250000	3.5236470000	23.0888490000
H	7.3029930000	3.3536170000	24.1670080000
H	6.1222940000	3.7508900000	22.8778230000
C	7.4669090000	5.7105920000	21.9163000000
C	8.1459010000	8.0355660000	21.3557610000
H	7.0832470000	8.0556030000	21.0960380000
H	8.3445320000	8.7641060000	22.1555220000
C	8.3662980000	8.4288150000	18.9001110000
C	9.0002100000	9.3490060000	16.6779320000
H	9.2663330000	10.4105960000	16.5504770000
H	7.9210520000	9.2236700000	16.5434390000
N	12.6094910000	0.8208980000	18.2034800000
C	11.8349960000	2.0249180000	17.6800110000
C	10.3260220000	1.6829760000	17.7896160000
C	9.4454900000	2.8813210000	17.5190980000
C	12.2817940000	3.2664400000	18.4527100000
C	8.9384190000	3.6377840000	18.5887320000
C	9.1747900000	3.2947230000	16.2048970000
C	8.1997880000	4.8007200000	18.3521450000
C	8.4346620000	4.4577160000	15.9663910000
C	7.9542060000	5.2180490000	17.0391900000
H	12.1231340000	2.1236830000	16.6271130000
H	10.1171720000	1.3035060000	18.7977500000
H	10.0944170000	0.8727290000	17.0849870000
H	13.3691910000	3.3987260000	18.3628380000
H	12.0259390000	3.2085490000	19.5172400000
H	11.8045140000	4.1572950000	18.0314780000
H	9.1333910000	3.3216730000	19.6133830000
H	9.5456590000	2.7068000000	15.3654320000
H	12.4016710000	-0.0300930000	17.6511310000
H	12.3523500000	0.6361560000	19.1943810000
H	7.8099180000	5.3812580000	19.1843490000
H	8.2207560000	4.7740220000	14.9499780000
H	7.3974870000	6.1329580000	16.8537660000
H	13.6224650000	0.9848820000	18.1594320000

- S-MET@CB[7]

XYZ file generated by gabedit : coordinates in Angstrom

O	6.9157860000	6.5962200000	14.4534290000
O	5.3844240000	3.2169400000	14.2007310000
O	7.6081030000	0.2922150000	14.4569920000
O	11.3943200000	0.0033000000	15.0323470000
O	14.2590340000	2.3022980000	15.4132650000
O	13.8664950000	5.9204500000	15.4382030000
O	10.6170430000	7.7448930000	14.9648880000
O	6.4658560000	7.0130380000	20.6146930000
O	5.7693740000	3.2879100000	20.3558820000
O	7.4473170000	-0.3031210000	20.5193800000
O	10.5456340000	-1.1537320000	21.0076210000
O	12.5310930000	1.9196090000	21.3010700000
O	12.2584940000	5.9848380000	21.3956730000
O	9.6019770000	8.3782170000	21.0715350000
N	7.0227210000	8.0237120000	16.3060260000

N	5.3220800000	6.5643260000	16.1593740000
N	4.5526540000	4.2424600000	16.1294200000
N	4.7393490000	2.0078850000	16.0923750000
N	6.0311530000	-0.0695220000	16.1416350000
N	7.9855340000	-1.1653310000	16.2492300000
N	10.3854900000	-1.4545750000	16.5594460000
N	12.5337200000	-0.8741210000	16.8704340000
N	14.1575370000	0.8992110000	17.2819970000
N	14.9436800000	2.9842920000	17.5399620000
N	14.7547490000	5.4198400000	17.5389380000
N	13.4972010000	7.2644210000	17.3171550000
N	11.5328270000	8.6572910000	16.9086520000
N	9.3156530000	8.8181250000	16.5862160000
N	6.6610610000	8.3702450000	18.7251270000
N	5.3127380000	6.5807750000	18.6251690000
N	4.5788180000	4.2552110000	18.5931680000
N	4.9811370000	2.0446740000	18.5402890000
N	6.1271790000	-0.1056340000	18.5993150000
N	7.7340290000	-1.6665890000	18.6452970000
N	10.1251960000	-2.1114810000	18.9218460000
N	12.1169180000	-1.1565720000	19.2831710000
N	13.6746600000	0.6791870000	19.6842800000
N	14.0989430000	2.8770690000	19.8498010000
N	13.8733700000	5.3115510000	19.8398720000
N	13.0816490000	7.4147630000	19.7459100000
N	11.1114680000	8.7955850000	19.3361140000
N	8.9358730000	9.2141900000	18.9951570000
C	6.4757740000	7.0094570000	15.5276160000
C	4.4171850000	5.5861880000	15.5826520000
H	4.6206780000	5.5236520000	14.5098410000
H	3.3862820000	5.9351530000	15.7431160000
C	4.9455130000	3.1614340000	15.3511000000
C	4.9259530000	0.6708290000	15.5549840000
H	5.1174150000	0.7742530000	14.4830570000
H	4.0045770000	0.0916950000	15.7131610000
C	7.2536770000	-0.2459460000	15.5045840000
C	9.2126060000	-1.7629610000	15.7518620000
H	9.0920280000	-2.8561630000	15.7061520000
H	9.3836090000	-1.3700170000	14.7453200000
C	11.4306790000	-0.6878180000	16.0485530000
C	13.8450640000	-0.3301470000	16.5690080000
H	13.8837130000	-0.1033710000	15.4991970000
H	14.5972870000	-1.0934000000	16.8140300000
C	14.4243360000	2.0889430000	16.6131420000
C	15.5347350000	4.2570160000	17.1605570000
H	16.5273640000	4.3351380000	17.6253960000
H	15.6360010000	4.2631090000	16.0715000000
C	14.0166300000	6.1705580000	16.6355250000
C	12.9289840000	8.4043320000	16.6068390000
H	13.0099280000	8.1937410000	15.5362260000
H	13.5094160000	9.3071820000	16.8488990000
C	10.4992340000	8.3318300000	16.0414670000
C	8.0842400000	8.8884020000	15.8116430000
H	7.7269510000	9.9298490000	15.8064810000
H	8.3149680000	8.5742850000	14.7896690000

C	6.1141950000	8.4319920000	17.3838760000
H	5.7069790000	9.4322210000	17.1800410000
C	5.0376840000	7.2994790000	17.3782610000
H	4.0034690000	7.6710330000	17.3700820000
C	3.9133480000	3.8228070000	17.3755190000
H	2.8584220000	4.1300950000	17.3854250000
C	4.1183960000	2.2716820000	17.3801080000
H	3.1844600000	1.7020410000	17.4818550000
C	5.9233730000	-0.8580970000	17.3527760000
H	4.9686180000	-1.4001630000	17.3827950000
C	7.1807430000	-1.7814010000	17.3071800000
H	6.9605830000	-2.8309390000	17.0707890000
C	10.8257940000	-2.3107990000	17.6617490000
H	10.7898560000	-3.3683360000	17.3669840000
C	12.2696170000	-1.7862470000	17.9651200000
H	13.0316800000	-2.5761900000	18.0085560000
C	14.6715190000	0.9095970000	18.6495550000
H	15.5040700000	0.2007130000	18.7586630000
C	15.0772320000	2.4019830000	18.8609340000
H	16.0981110000	2.5353210000	19.2445240000
C	14.7503520000	5.9881810000	18.8718720000
H	15.7729260000	6.0593540000	19.2678160000
C	14.0525550000	7.3717890000	18.6722890000
H	14.7375530000	8.2287720000	18.7366270000
C	11.0880110000	9.4617810000	18.0352680000
H	11.6538460000	10.4027440000	18.0781230000
C	9.5589430000	9.6594480000	17.7606460000
H	9.2803140000	10.7006310000	17.5450070000
C	6.1878320000	7.2838510000	19.4469700000
C	4.4094280000	5.5790370000	19.1642890000
H	4.6000380000	5.5113100000	20.2394120000
H	3.3691030000	5.8958560000	18.9918290000
C	5.1810890000	3.2121030000	19.2763740000
C	5.0914380000	0.7393520000	19.1722760000
H	5.3382170000	0.8923480000	20.2267580000
H	4.1214000000	0.2232200000	19.0895480000
C	7.1376930000	-0.6520610000	19.3735860000
C	8.7634930000	-2.5441120000	19.1815310000
H	8.6369690000	-2.5955880000	20.2665820000
H	8.6195590000	-3.5409300000	18.7473090000
C	10.8906070000	-1.4405920000	19.8546560000
C	13.2295980000	-0.6500560000	20.0705850000
H	12.9017290000	-0.6157760000	21.1135010000
H	14.0847310000	-1.3345010000	19.9780830000
C	13.3485780000	1.8339420000	20.3719180000
C	14.2999310000	4.1280400000	20.5720450000
H	15.3692690000	4.2192440000	20.8218630000
H	13.7129180000	4.0819800000	21.4930660000
C	12.9806640000	6.2121760000	20.4263670000
C	12.3273510000	8.6017030000	20.1078850000
H	12.0366840000	8.5106520000	21.1587090000
H	12.9828450000	9.4748340000	19.9797150000
C	9.8532890000	8.7426150000	19.9235410000
C	7.5270560000	9.3847090000	19.2987740000
H	7.2001620000	10.3669820000	18.9316580000

H	7.4194530000	9.3414620000	20.3866800000
N	9.5858930000	1.5018180000	21.3816830000
C	9.2165050000	2.4643860000	20.2532040000
C	9.8431100000	1.8856030000	18.9549810000
C	9.7079210000	2.8365970000	17.7876000000
C	9.6711120000	3.8834930000	20.5957620000
C	8.4726360000	3.0032260000	17.1413660000
C	10.7973290000	3.6296550000	17.3919280000
C	8.9551760000	1.7729610000	22.7172570000
C	8.3229780000	3.9679610000	16.1408100000
C	10.6495750000	4.5937860000	16.3901550000
C	9.4068310000	4.7731870000	15.7707570000
H	8.1222060000	2.4302960000	20.1827540000
H	9.3576490000	0.9273500000	18.7234600000
H	10.9057870000	1.6751450000	19.1287080000
H	9.4165830000	4.5548330000	19.7702370000
H	10.7543550000	3.9398730000	20.7589400000
H	9.1599780000	4.2609260000	21.4886670000
H	10.6226220000	1.4907880000	21.4664820000
H	7.6231780000	2.3850530000	17.4302960000
H	11.7606130000	3.5062200000	17.8863640000
H	9.2125420000	0.9453480000	23.3855130000
H	7.8710490000	1.8272590000	22.5837100000
H	9.3397240000	2.7104840000	23.1239430000
H	7.3666240000	4.1017380000	15.6454640000
H	11.4957920000	5.2080120000	16.0922890000
H	9.2801530000	5.5456930000	15.0168170000
H	9.3071570000	0.5457460000	21.0962410000

- R-MET@CB[7]

XYZ file generated by gabedit : coordinates in Angstrom

O	7.3988240000	7.2398970000	14.1648680000
O	6.7026440000	3.5275310000	13.7302640000
O	8.4265840000	-0.0151700000	13.6353840000
O	11.5717500000	-0.8873030000	13.9584070000
O	13.4913320000	2.1065720000	14.6562740000
O	13.2006810000	6.1553330000	14.9739740000
O	10.5370040000	8.5712520000	14.8097090000
O	6.0924110000	5.9996370000	20.0877880000
O	4.5683370000	2.6627830000	19.4480750000
O	6.7496440000	-0.2608810000	19.4961340000
O	10.5553040000	-0.4836470000	20.0021620000
O	13.4659800000	1.7911700000	20.7817740000
O	13.0822590000	5.3334930000	21.0902240000
O	9.7742320000	7.1183570000	20.8146040000
N	7.0373530000	8.3320770000	16.1964040000
N	5.7348640000	6.5836830000	15.6722030000
N	5.0461150000	4.2939650000	15.1895170000
N	5.4325470000	2.0846060000	15.0601650000
N	6.5884770000	-0.0604400000	15.0798270000
N	8.1790260000	-1.6208810000	15.3108950000
N	10.5516980000	-2.0720090000	15.6912720000
N	12.5715010000	-1.1789780000	16.0457470000
N	14.1508730000	0.6559510000	16.3637780000

N	14.5722940000	2.8487040000	16.5960890000
N	14.3251400000	5.2701610000	16.8272650000
N	13.5053810000	7.3609840000	16.9486080000
N	11.4771340000	8.7189850000	16.9449440000
N	9.2879250000	9.1341670000	16.7015570000
N	6.7092980000	7.6519280000	18.5479730000
N	5.0459110000	6.2408220000	18.0149020000
N	4.3208000000	3.9591870000	17.5191870000
N	4.4949600000	1.7331040000	17.3074440000
N	5.7657830000	-0.3558630000	17.3799150000
N	7.6908310000	-1.4566950000	17.7167810000
N	10.0827680000	-1.7437010000	18.0897230000
N	12.2074600000	-1.1221350000	18.4803240000
N	13.8926630000	0.6088930000	18.8086920000
N	14.7631090000	2.6617970000	19.0459380000
N	14.5351810000	5.0835780000	19.2785070000
N	13.2187050000	6.8986020000	19.3572720000
N	11.1908180000	8.2595910000	19.3518410000
N	8.9718180000	8.4220620000	19.0453570000
C	6.7992960000	7.3634150000	15.2324550000
C	5.0355670000	5.6839540000	14.7702990000
H	5.5336570000	5.7465230000	13.7988120000
H	3.9877620000	6.0057490000	14.6699610000
C	5.8285410000	3.3296040000	14.5746110000
C	5.7478940000	0.8652650000	14.3359350000
H	6.2902350000	1.1414330000	13.4271460000
H	4.8080620000	0.3586610000	14.0655300000
C	7.7984700000	-0.5104910000	14.5792780000
C	9.3384060000	-2.4357750000	14.9830270000
H	9.0938990000	-3.4812190000	15.2093460000
H	9.5402770000	-2.3290050000	13.9132110000
C	11.5628160000	-1.3294830000	15.1131810000
C	13.8619110000	-0.6082700000	15.7035480000
H	13.8696430000	-0.4447710000	14.6217260000
H	14.6573470000	-1.3166080000	15.9739710000
C	14.0143750000	1.8925560000	15.7598520000
C	14.9526340000	4.1707070000	16.1107310000
H	16.0490540000	4.2664530000	16.1803190000
H	14.6463260000	4.2427070000	15.0627340000
C	13.6190420000	6.2515870000	16.1261640000
C	12.8660250000	8.5978730000	16.5358590000
H	12.8895530000	8.6428510000	15.4426840000
H	13.4432120000	9.4357150000	16.9524810000
C	10.4410860000	8.7722390000	16.0198450000
C	8.0240940000	9.3848850000	16.0338450000
H	7.5985370000	10.3212680000	16.4196230000
H	8.2313070000	9.4860530000	14.9646370000
C	6.1351690000	8.2285270000	17.3270700000
H	5.6780260000	9.2042300000	17.5436960000
C	5.1108440000	7.1381750000	16.8761950000
H	4.1131590000	7.5361490000	16.6432320000
C	4.0639360000	3.7275810000	16.0987150000
H	3.0536880000	4.0685150000	15.8321420000
C	4.2709870000	2.1837500000	15.9433150000
H	3.4091930000	1.6633980000	15.5016770000

C	6.0443720000	-0.9711220000	16.0979360000
H	5.1562250000	-1.4901260000	15.7128980000
C	7.2553080000	-1.9098560000	16.3937430000
H	7.0023190000	-2.9786030000	16.4092200000
C	10.8471080000	-2.4536130000	17.0651720000
H	10.7441900000	-3.5404650000	17.1900810000
C	12.3083760000	-1.9276930000	17.2779950000
H	13.0525640000	-2.7257380000	17.4053490000
C	14.7939390000	0.7415270000	17.6647790000
H	15.6188890000	0.0185760000	17.7311810000
C	15.2472960000	2.2325960000	17.7500300000
H	16.3346430000	2.3730480000	17.6715170000
C	14.8861630000	5.8099960000	18.0755870000
H	15.9768150000	5.9177080000	17.9904900000
C	14.1319480000	7.1677570000	18.2398410000
H	14.7899320000	8.0146060000	18.4802120000
C	11.0717090000	9.2114110000	18.2600900000
H	11.6094590000	10.1396010000	18.5017210000
C	9.5249290000	9.4023050000	18.1096010000
H	9.1771930000	10.4123490000	18.3697920000
C	5.9718130000	6.5618900000	18.9993100000
C	4.0253650000	5.2223700000	18.1813520000
H	3.9266200000	5.0135970000	19.2504410000
H	3.0757000000	5.6181280000	17.7931860000
C	4.4780430000	2.7736210000	18.2242350000
C	4.5252980000	0.3333070000	17.6957260000
H	4.3840930000	0.2924710000	18.7799010000
H	3.7025500000	-0.1935050000	17.1924010000
C	6.7432410000	-0.6402730000	18.3271090000
C	8.7319090000	-2.1334230000	18.4702930000
H	8.5925580000	-1.8790080000	19.5257020000
H	8.6292810000	-3.2218900000	18.3365070000
C	10.9073940000	-1.0423830000	18.9665880000
C	13.3577880000	-0.6715890000	19.2432280000
H	13.0523720000	-0.5562110000	20.2874350000
H	14.1423750000	-1.4404310000	19.1724170000
C	13.9735540000	1.7008920000	19.6656810000
C	15.2056410000	3.8738790000	19.7139030000
H	16.2853170000	3.9918130000	19.5425900000
H	15.0137320000	3.7520800000	20.7839740000
C	13.5535080000	5.7250380000	20.0219980000
C	12.4446110000	7.9472460000	20.0107550000
H	12.2163660000	7.5980580000	21.0218000000
H	13.0504690000	8.8641450000	20.0637080000
C	9.9582700000	7.8428780000	19.8369790000
C	7.5710580000	8.4165020000	19.4384360000
H	7.2095330000	9.4558530000	19.4837020000
H	7.5061880000	7.9610310000	20.4309280000
N	10.6913430000	1.8278100000	13.7332070000
C	10.0070260000	2.6111410000	14.8551600000
C	10.2625140000	1.8219330000	16.1682770000
C	9.7843280000	2.5772340000	17.3874560000
C	10.5035800000	4.0565750000	14.8783630000
C	10.6946100000	3.3029940000	18.1724790000
C	8.4176590000	2.6311020000	17.7031830000

C	10.4693720000	2.3410160000	12.3386080000
C	10.2465770000	4.1006720000	19.2302690000
C	7.9668560000	3.4235480000	18.7631990000
C	8.8782880000	4.1718690000	19.5177700000
H	8.9384670000	2.5905730000	14.6109100000
H	11.3370120000	1.6207450000	16.2655340000
H	9.7550500000	0.8502720000	16.1061810000
H	10.2441180000	4.5867600000	13.9551640000
H	11.5891900000	4.1190510000	15.0226950000
H	10.0192330000	4.5880370000	15.7031590000
H	10.3457250000	0.8517830000	13.7635760000
H	11.7587080000	3.2592120000	17.9383520000
H	7.7035720000	2.0563120000	17.1130720000
H	10.9029560000	1.6189590000	11.6403510000
H	10.9583800000	3.3097120000	12.2201110000
H	9.3929570000	2.4303690000	12.1685770000
H	10.9552890000	4.6692420000	19.8289190000
H	6.9094030000	3.4620030000	19.0093570000
H	8.5217970000	4.8202050000	20.3140720000
H	11.7095170000	1.7891410000	13.9428830000
